# Post-Traumatic Growth Following Exposure to Memorial Reports of the 5.12 Wenchuan Earthquake: The Moderating Roles of Self-Esteem and Long-Term PTSD Symptoms

**DOI:** 10.3390/ijerph16183239

**Published:** 2019-09-04

**Authors:** Zhihao Ma, Yiwei Xia, Zhongxuan Lin

**Affiliations:** 1 Computational Communication Collaboratory, School of Journalism and Communication, Nanjing University, Nanjing 210023, China; 2 School of Law, Southwestern University of Finance and Economics, Chengdu 611130, China; 3 School of Journalism and Communication, Jinan University, Guangzhou 510632, China

**Keywords:** post-traumatic growth, media exposure, memorial reports, self-esteem, PTSD symptoms, Wenchuan

## Abstract

Media exposure during a traumatic event has been found to be associated with negative psychological consequences. However, the post-disaster role of the mass media and the possible positive psychological consequences of media exposure has received less attention. In the present study, we hypothesized that exposure to memorial media reports would lead to improved post-traumatic growth (PTG). Further, we evaluated the moderating role of self-esteem and long-term post-traumatic stress disorder (PTSD) symptoms in the relationship between media exposure and PTG. Using a cross-sectional design, we surveyed individuals (*N* = 1000, mean age = 45.62, 43.5% male) who were recruited from disaster-affected communities ten years after the 5.12 Wenchuan earthquake which was the largest country-level trauma in the past three decades. Results revealed that individuals with lower self-esteem or lower PTSD symptoms would have higher psychological growth with greater exposure to memorial news reports. For individuals who reported having both high levels of self-esteem and PTSD symptoms, the relationship between media exposure and PTG was negative. These findings help present trauma in a new light, particularly regarding the rapid and instantaneous new coverage of the digital age. This study also has multi-disciplinary, cross-cultural, and clinical implications for the fields of psychology, public health, and communications.

## 1. Introduction

Previous studies have demonstrated that media exposure during traumatic events can lead to negative psychopathological responses, such as anxiety [1,2], depression [2], and post-traumatic stress disorder (PTSD) symptoms [2,3]. Previous research reported that American adults who had extensive exposure to television news coverage of the 9/11 terrorist attacks on the day of the attack and the next day reported higher PTSD symptoms than those with less exposure [4]. Bodas and colleagues reported that a Jewish sample in Israel reported severe anxiety, sleep disorder, and a variety of adverse psychological outcomes with increased frequency of viewing newscasts during the war [1]. Further, the relationship between media exposure during traumatic events and psychopathological consequences could be persistent for some individuals [5]; however, the effects of media exposure following traumatic events with regards to long-term post-traumatic adaptation have not been previously studied. 

In recent years, research on the positive psychological outcomes associated with media exposure has emerged [5,6], and post-traumatic growth (PTG), the psychological growth resulting from exposure to a traumatic incident, has been observed and examined as the key indicator following exposure to traumatic incidents [7]. Yu and colleagues found that adolescents reported increased PTG and reduced suicidal ideation one month after the 5.12 Wenchuan earthquake if they viewed encouraging and touching earthquake-related news reports during the earthquake [6]. Park and colleagues reported that American adults who had extensive exposure to television reports during the 9/11 terrorist attacks reported increased levels of PTG [5]. He and his colleagues proposed two competing mechanisms for these traumatic-related media-effects [4]. First, media exposure may play a role as a type of secondary traumatization, which can trigger individuals to re-experience the traumatic event. Second, individuals may experience stress alleviation when they attempt to seek more information about the traumatic event, as this serves as a coping strategy to alleviate psychological stress. Thus, media coverage about disasters may play an important role in reconstructing individuals’ mental health [6]. 

Previous research on the association between media exposure and positive psychological changes were only assessed during or immediately following the traumatic event, and the potential long-term associations have largely been ignored. The mass media routinely delivers news coverage about historically significant traumatic events via memorial reporting; thus, developing a framework to verify the influence of these memorial reports on the promotion of individuals’ PTG would be beneficial. 

On 12 May 2008, an earthquake with a magnitude of 8.0 on the Richter scale hit Wenchuan, in Sichuan province, China. As of 25 September 2008, the global death toll for this disaster was estimated to be 69,227, with 374,642 injuries and 17,923 missing persons [8]. For all of China, the 5.12 Wenchuan earthquake has been viewed as not only the most grievous natural disaster in the past three decades but also an indelible national trauma. National and local media organizations have extensively reported on the events related to the earthquake emphasizing post-disaster rebuilding and commemorative activities as a part of China’s centralized propaganda strategy [9,10]. 

Memorial reporting on the 5.12 Wenchuan earthquake has been rising with the goal of constructing a national memory of overcoming this natural disaster [11]. Exposure to memorial media coverage may have positive psychological consequences on those individuals who experienced the disaster. Researchers in the field of communication studies have developed the cultivation theory to explain the long-term effects of media exposure [12]. The cultivation theory assumes that media consumption behavior influences the reconstruction of viewers’ conceptualization of social reality [13]. Research has demonstrated that people who grew up with heavy exposure to television violence viewed the world as being more dangerous than those who were light television viewers [14]. Thus, in the context of the 5.12 Wenchuan earthquake, it is reasonable to theorize that individuals’ exposure to uplifting content via mass media may contribute to positive psychological changes. Given this body of research, we proposed our first research hypothesis as:

**Hypothesis** **1.** *Exposure to post-disaster memorial reports of 5.12 Wenchuan earthquake would be associated with PTG.*

Effects of media exposure on individuals’ psychological outcomes have been shown to be context-dependent [4,15]. A recent review summarized research suggesting that the relationship between media exposure during a disaster and viewers’ short-term cognitive and emotional responses may be moderated by both developmental factors and sympathetic reactivity [16]. Researchers need to explore potential moderators to help identify those who are susceptible to the psychological effects of media exposure and to expand the field’s understanding of long-term psychological growth in the post-disaster period. Based on existing research [17,18,19], we identified self-esteem and PTSD symptoms as being potential moderating factors in the theoretical framework of the present study. 

Self-esteem, a key aspect of the self-system, has been researched in numerous psychological and mental health studies [20] and plays a crucial role in mental health adaptation [21]. Terror management theory has indicated that self-esteem functions as a buffer for intrapersonal cultural anxiety when individuals are exposed to a traumatic event [22]. Previous studies have demonstrated that self-esteem is positively associated with PTG in adolescents [18], decreased suicidal ideation in college students [23], and PTSD symptoms in disaster survivors [19]. Thus, survivors with low self-esteem who experienced the 5.12 Wenchuan earthquake may show limited long-term psychological growth, and exposure to memorial media coverage may serve as a source for vicariously improving their self-esteem and directly enhancing their psychological growth. On the other hand, those survivors with high inherent self-esteem may not experience the same psychological benefits of long-term media exposure due to the repetitive and homogeneous memorial news reports. Therefore, we proposed the second hypothesis:

**Hypothesis** **2.** *Self-esteem moderated the relationship between media exposure and PTG. Specifically, the relationship between media exposure to post-disaster memorial reports of the 5.12 Wenchuan earthquake and PTG would be stronger for survivors with lower self-esteem than for survivors with higher self-esteem.*

The second potential moderator in the relationship between media exposure and PTG is the presence of long-term PTSD symptoms, which are also critical during survivors’ post-disaster adaptation [24,25]. The relationship between PTG and PTSD symptoms has been found to be complex, and previous research has provided contradictory evidence [24,26]. For example, Hafstad and colleagues found that PTG was positively associated with PTSD symptoms in children 30 months after a disaster [24], while Wu and colleagues found no relationship between PTG and PTSD symptoms in children 18 months after a disaster [26]. One explanation might be that PTG and PTSD are the results of different mechanisms [26]; however, previous research has also shown that stressors work as catalysts for individuals’ long-term psychological changes [25]. The presence of distress symptoms in those who experienced the 5.12 Wenchuan earthquake survivors may be sustained for an extended period [4], and the potential psychological benefits from media exposure may be weakened in those with numerous PTSD symptoms. Therefore, we hypothesized:

**Hypothesis** **3.** *Long-term PTSD symptoms would be another moderator of the relationship between media exposure and PTG. Specifically, the relationship between media exposure to post-disaster memorial reports of 5.12 Wenchuan earthquake and PTG would be stronger for survivors with lower PTSD symptoms than for survivors with higher PTSD symptoms.*

## 2. Materials and Methods 

### 2.1. Sampling Procedures

The current study analyzed the data collected by and reported in a previous study [19]. In the original study, 1000 adult survivors (M_age_ = 45.62, SD = 11.63; 43.5% male; 82.7% married; 55.7% living in rural areas) were recruited from six counties in Sichuan in May 2018, on the tenth anniversary of the 5.12 Wenchuan earthquake. The whole sampling procedure had two steps. First, the survey team selected 26 communities, based on the degree of damage caused by the earthquake and residents’ subsequent living arrangements. Second, 30 to 50 individuals were selected from each settlement according to local population size. The inclusion criteria included being older than 18 years, residing in the local county before the earthquake, and not migrating to other areas more than one year after the earthquake.

### 2.2. Measures

#### 2.2.1. Post-Traumatic Growth Inventory

PTG was assessed using the post-traumatic growth inventory (PTGI) [7]. This instrument consists of 21 items with a six-point Likert scale (from 1 = no change to 6 = a high degree of change) and assesses changes in five PTG domains: relating to others, new possibilities, personal strength, appreciation of life, and spiritual change. This inventory has shown good reliability in several countries [7,27,28]. In the present study, the PTGI was translated into Chinese and then back-translated into English by two bilingual researchers. Our pilot study suggested one item needed to be revised from “I have a stronger religious faith” to “I have a stronger faith” for the Chinese context since most Chinese people hold traditional Chinese values (e.g., Confucianism, Taoism, or the Yin-Yang system) rather than specific religious beliefs [29,30]. In the present study, Cronbach’s alpha for the PTGI was 0.928. The overall score of PTGI was calculated as means of these 21 items.

#### 2.2.2. Exposure to Memorial Reports

Since the survey was carried out in developing areas of China, most participants did not have adequate media literacy to distinguish between media types and provide detailed information on these sources. Our pilot survey indicated that sophisticated measurement of media usage would not be feasible. Hence, based on methods used in previous research [31,32], the degree of exposure to memorial reporting of the earthquake (i.e., media exposure) was measured using a single item. All participants were asked to estimate how much time they spent watching memorial reports of the 5.12 Wenchuan earthquake via media platforms (e.g., newspaper, broadcasting, television, Internet) in the previous one month. Responses ranged from 1 (never) to 5 (a lot).

#### 2.2.3. Rosenberg Self-Esteem Scale

The Rosenberg self-esteem scale was used to assess self-esteem [33] and is a widely used 10-item instrument with ratings ranging from 1 (strongly agree) to 4 (strongly disagree), and provides an overall evaluation of one’s self-worth or value. Cronbach’s alpha for the present study is 0.668. The overall score of self-esteem was calculated as means of 10 items.

#### 2.2.4. PTSD Check List-Civilian Version

The PTSD Check List–Civilian Version (PCL-C) was used to assess PTSD symptoms [34]. This instrument consists of 17 items that were developed according to the diagnostic criteria of PTSD set by the Diagnostic and Statistical Manual of Mental Diseases, fourth edition [34,35]. In this study, each item was measured from 1 (not at all bothered) to 5 (extremely severe). The PCL-C overall score was calculated by the mean of 17 items, and the Cronbach’s alpha in this study is 0.898. In addition, we also used the sum score of 17 the items to identify the individuals who were experiencing PTSD. Given the previously established cutoff score of 50 [34], 231 participants were identified as being PTSD survivors.

#### 2.2.5. Control Variables

We included demographic characteristics, socioeconomic status, and direct earthquake exposure as control variables. The descriptive statistics of these control variables have been reported elsewhere [4].

### 2.3. Ethical Approval and Informed Consent

The study was approved by the ethics committee of the Department of Sociology and Social Work, Sun Yat-sen University. Before the survey, all participants received written information about the study and signed a consent form if they volunteered to participate in the study. In addition, all data were coded to preserve participants’ anonymity.

### 2.4. Statistical Analysis

We calculated descriptive statistics in the present study. Main effects of exposure to memorial reports, self-esteem, and long-term PTSD symptoms on PTG were estimated via the Tobit regression model. As the dependent variables in the present study are truncated with definite boundaries (i.e., ranging from 1 to 6), ordinary least squares (OLS) regression yields biased estimates as the assumption of homoscedasticity is violated. To reduce possible bias, we adopted the Tobit regression model, which is designed for censored response [36], to estimate the coefficients. To test for moderation, we added multiplicative terms of self-esteem × exposure to the memorial reports and PCL-C score × exposure to memorial reports in a two-way interaction estimation model. We also explored three-way interaction effects of media exposure and two moderation variables (self-esteem × PCL-C score × exposure to memorial reports), then simple slopes were computed and plotted to further demonstrate the moderation effects [37].

## 3. Results

### 3.1. Descriptive Statistics

Means and standard deviations are reported in Table 1. The mean score for PTGI was 3.1, which indicates that the participants exhibited a moderated level of PTG. The PCL-C score was less than 3, which is the average score of the cut-off point to identify PTSD. Participants showed a relatively higher self-esteem status and moderate media exposure to memorial news coverage.

### 3.2. Main Effects 

As displayed in Model 1 of Table 2, participants with higher PTSD symptoms reported higher PTG (*B* = 0.66, *p* < 0.001). This result verified that the long-term PTSD symptoms still work as the catalyst for nurturing PTG. The coefficient of self-esteem in Model 1 was also positively significant (*B* = 0.11, *p* < 0.05). 

The coefficient of exposure to memorial reports and PTG in Model 1 was significantly positive (*B* = 0.08, *p* < 0.001). This finding indicates that those participants who were exposed to more memorial reports about the 5.12 Wenchuan earthquake showed greater positive psychological changes than those with less media exposure. Thus, Hypothesis 1 was statistically supported.

### 3.3. Moderation Effects

Model 2 showed the results of the Tobit estimates of PTG with two two-way interactions to evaluate Hypothesis 2 and Hypothesis 3 (Table 2). The interaction between self-esteem and exposure to memorial reports was negatively significant (*B* = −0.16, *p* < 0.01), indicating that those with lower self-esteem have a stronger association between psychological growth and media exposure than those with higher self-esteem. The interaction between PCL-C scores and exposure to memorial reports also was shown to be significantly negative (*B* = −0.15, *p* < 0.001), indicating that survivors with less PTSD symptoms received greater psychological benefits from exposure to memorial media coverage, which statistical supports Hypothesis 2 and Hypothesis 3. Both self-esteem and PTSD symptoms eroded the positive association between media exposure and psychological growth. Simple slopes analysis for the two-way interactions revealed that the positive association between media exposure and psychological growth are significant for those individuals with fewer PTSD symptoms (slope coefficient = 0.18, *p* < 0.001) and those with lower self-esteem (slope coefficient = 0.14, *p* < 0.001). However, the relationship between media exposure and psychological growth was not significant for survivors with more PTSD symptoms (slope coefficient = −0.02, *p* > 0.05) or higher self-esteem (slope coefficient = 0.02, *p* > 0.05). 

Model 3 estimated the effects of a potential three-way interaction (Table 2). The three-way interaction was found to be negatively significant (*B* = −0.06, *p* < 0.05) indicating that fewer PTSD symptoms and lower self-esteem may contribute to greater positive psychological changes in survivors who expose to memorial reports in the post-disaster era.

**Figure 1:**
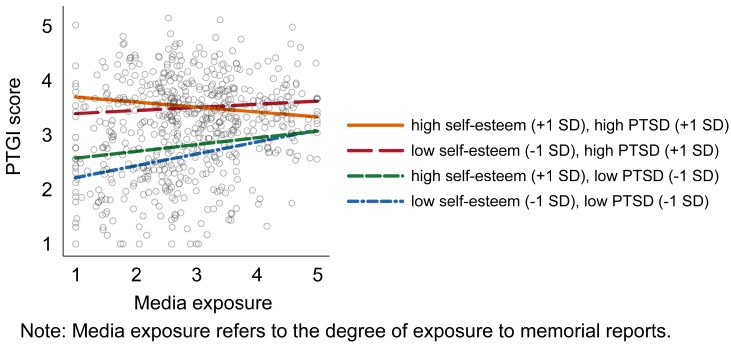
PTG as a function of self-esteem and PTSD symptoms across different degrees of exposure to memorial reports.

To examine the three-way interactions [38], we plotted the moderation effects of the three-way interaction (Figure 1). Overall, those participants with more PTSD symptoms reported greater PTG than those who reported with fewer PTSD symptoms. The number of PTSD symptoms significantly moderated the associations among self-esteem, media exposure and PTG. For participants with high PTSD symptoms, the relationship between media exposure and PTG for those individuals who also had low self-esteem was positive. The direction of this relationship was reversed for those individuals who had high self-esteem. Individuals with greater media exposure to memorial reports reported lower PTG than those with less media exposure did. However, for those reporting fewer PTSD symptoms, the association between media exposure and PTG was attenuated if they also reported higher levels of self-esteem. Further, individuals with low self-esteem reported higher PTG in the context of greater media exposure.

Next, we conducted a slope difference test using the guidelines of Dawson and Richter (2006) to evaluate the three-way interactions (Table 3). Participants with more PTSD symptoms and higher self-esteem showed a significantly attenuated association between PTG and media exposure (*B* = −0.09, *p* < 0.05); whereas, individuals with lower self-esteem did not demonstrate a significant association between the degree of exposure to memorial reports and PTG (*B* = 0.06, *p* > 0.05). Individuals who reported fewer PTSD symptoms and higher self-esteem (*B* = 0.12, *p* < 0.01) had relatively less positive psychological changes following media exposure when compared to individuals with lower self-esteem (*B* = 0.22, *p* < 0.001). These results suggest that the positive effect of media exposure following a disaster was the strongest for those individuals with low self-esteem and few long-term PTSD symptoms. Exposure to memorial media reports was associated with negative psychological consequences in those participants with high self-esteem and high long-term PTSD symptoms.

## 4. Discussion

The present study examined the role of memorial news coverage following natural disasters and found that media exposure has different psychological effects among survivors depending on their individual characteristics and psychological traits. The negative influences of media exposure were significant among the survivors who reported more PTSD symptoms and higher self-esteem. Media exposure was positively associated with psychological growth only among survivors with lower PTSD symptoms or lower self-esteem. These results are novel as previous research on PTG following traumatic events has focused on personality traits [39], psychological determinants [18], mental complications [40,41], and short-term media effects during traumatic events [6]. 

To the best of our knowledge, this is the first study to link media consumption of memorial reports of a natural disaster and positive psychological growth (PTG) in long-term post-disaster adaptation. Our results suggest that there might be psychological benefits from viewing memorial media reports for survivors with unexpressed pathology or lower self-esteem. The mass media, as a governance-assistant institution in China, provides extrinsic emotional support to foster healing from grief following disasters. The media-constructed social-reality presented the positive side of post-disaster rebuilding, which has been linked to decreased traumatic reactions among participants and potentially could strengthen individuals’ mental state [42]. 

Further, self-esteem was found to be a crucial psychological determinant for participants’ PTG. This positive association is consistent with previous research [18] that has demonstrated that people with enhanced self-esteem tend to report higher PTG than people with lower self-esteem. These findings support the terror management theory that proposes that self-esteem is an anxiety buffer for individuals during their post-disaster adaptation [22]. In addition, our data demonstrated that even ten years after the disaster, long-term PTSD symptoms could influence individuals’ psychological changes. This relationship is consistent with most of the previous research [25,43], which has suggested that long-term traumatic reactions may result in reframing individuals’ worldview about post-disaster adaption, leading to more PTG. 

Results of the two-way interactions suggest that self-esteem and PTSD symptoms moderated the positive relationship between exposure to memorial reports and PTG. For participants, media exposure to the disaster represents a vicarious source of traumatization [4,22]. Regardless of the media’s framing of the event, the repeated and periodic memorial reports toward such traumatic events appear to contribute to secondary traumatization following the disaster. People with high PTSD symptoms are a population susceptible to stress reactions. For those with high self-esteem, PTG mostly developed via some inherent and active coping strategies rather than passive information exposure. It is noticeable; however, that results of the three-way interaction demonstrated that participants with both high self-esteem and high PTSD symptoms reported lower PTG with increased media exposure. These findings illustrate the potential negative influences of media use on psychological changes in post-disaster adaption.

Understanding the role of mass media in the years following a disaster also has significant practical implications for both the mental health promotion activities and publicity regulation surrounding disaster-related information. PTG is an important factor for post-traumatic conditions, including loneliness, depression, and resilience [41]. This study contributes to a growing body of research focused on ways to effectively adapt communication strategies for developing PTG and avoiding unnecessary exposure to long-term traumatization.

Although the present study provided important contributions to the literature by examining the direct effect of exposure to memorial reports on PTG and its moderation by self-esteem and long-term PTSD symptoms in the post-disaster adaption with data collected from six diverse counties that experienced the 5.12 Wenchuan earthquake, it has several limitations. First, this cross-sectional design cannot make inferences regarding the causal relationship. There is no current agreement in the field of research about the direction of the relationship between media effects and psychological reactions in the natural disaster scenarios [4]. Longitudinal designs were needed to test for causality. Second, the non-random sampling in our data may weaken the generalizability of the present study’s results. Third, gender differences were not discussed in the present study. Previous studies suggest that women may report more positive psychological changes compared to men [44,45]. These potential variations of media effects should be taken into consideration in future research. In addition, our data focused on the long-term media effects on PTG for direct disaster survivors. Since the mass media deliver memorial reports for the entire country, those not directly experiencing the earthquake may also have psychological reactions toward the media coverage [13]. Future studies should examine the media effects of disaster-related memorial reports on non-victims.

## 5. Conclusions

As we are in a period of rapid, global dissemination of news, as well as extensive, repetitive media exposure to disasters, research on how individual characteristics contribute to different responses to this media exposure is critical. Our study shows that media exposure to memorial reports has different influences on survivors’ psychological growth, depending on their individual characteristics and psychological traits. The relationship between media exposure and PTG was negative in those individuals who reported high levels of both self-esteem and PTSD symptoms. For survivors with lower PTSD symptoms or lower self-esteem, media exposure had a positive association with PTG. These findings may provide a new perspective on the field of trauma research.

## Figures and Tables

**Table 1:** Descriptive statistics.

Variable	Obs	Mean	Std. Dev.	Min	Max
PTGI score	1000	3.109	0.809	1	5.143
PCL-C score	1000	2.364	0.626	1	4.412
PTSD survivors ^a^	1000	0.231	0.422	0	1
Self-esteem	1000	2.883	0.391	1.7	4
Exposure to memorial reports	1000	2.837	0.963	1	5

Note: PTGI refers to post-traumatic growth inventory; PCL-C refers to The PTSD Check List–Civilian Version; PTSD refers to post-traumatic stress disorder. ^a^ Participants with a summed score of 17 PCL-C items ≥ 50 were identified as PTSD survivors.

**Table 2:** Tobit regression model of exposure to memorial reports, self-esteem, and PTSD symptoms on post-traumatic growth inventory (PTG).

Variables	Model 1	Model 2	Model 3
	B (Std. Err.)	B (Std. Err.)	B (Std. Err.)
Interaction Item			
Self-esteem × PCL-C score × exposure to memorial reports			−0.06 *
			(0.03)
PCL-C score × exposure to memorial reports		−0.15 ***	0.01
		(0.03)	(0.09)
Self-esteem × exposure to memorial reports		−0.16 **	−0.02
		(0.05)	(0.09)
Independent variables			
Exposure to memorial reports	0.08 ***	0.91 ***	0.49 +
	(0.02)	(0.18)	(0.27)
PCL-C score	0.66 ***	1.08 ***	1.07 ***
	(0.03)	(0.10)	(0.10)
Self-esteem	0.11 *	0.57 ***	0.58 **
	(0.06)	(0.17)	(0.17)
Control variables	Yes	Yes	Yes
Constant	0.47 +	−1.78 **	−1.76 **
	(0.26)	(0.56)	(0.56)
Observations	1000	1000	1000
Degree of freedom (DF)	30	32	33
chi2	496.7	522.4	526.5
Log likelihood	−961	−948.1	−946
Pseudo R-squared (PR2)	0.205	0.216	0.218

*** *p* < 0.001, ** *p* < 0.01, * *p* < 0.05, + *p* < 0.1; detailed coefficients of all control variables are reported in Table A1 in Appendix A.

**Table 3:** Slope difference test of three-way interactions.

	Slope	Delta-Method Std. Err.	Z Value	*p*-Value	95% Conf. Interval
High self-esteem (+1 SD), high PTSD (+1 SD)	−0.091	0.039	−2.33	0.020	−0.168 to −0.015
Low self-esteem (−1 SD), high PTSD (+1 SD)	0.057	0.036	1.60	0.109	−0.013 to 0.127
High self-esteem (+1 SD), low PTSD (−1 SD)	0.124	0.038	3.31	0.001	0.051 to 0.198
Low self-esteem (−1 SD), low PTSD (−1 SD)	0.218	0.040	5.44	0.000	0.139 to 0.296

## Appendix A

**Table A1:** Tobit regression model with coefficients of all control variables.

Variables	Model 1	Model 2	Model 3
	B (Std. Err.)	B (Std. Err.)	B (Std. Err.)
Interaction Item			
Self-esteem × PCL-C score × exposure to memorial reports			−0.06 *
			(0.03)
PCL-C score × exposure to memorial reports		−0.15 ***	0.01
		(0.03)	(0.09)
Self-esteem × exposure to memorial reports		−0.16 **	−0.02
		(0.05)	(0.09)
Independent variables			
Exposure to memorial reports	0.08 ***	0.91 ***	0.49 +
	(0.02)	(0.18)	(0.27)
PCL-C score	0.66 ***	1.08 ***	1.07 ***
	(0.03)	(0.10)	(0.10)
Self-esteem	0.11 *	0.57 ***	0.58 ***
	(0.06)	(0.17)	(0.17)
Demographic and socioeconomic variables			
Male	0.10 *	0.10 *	0.10 *
	(0.04)	(0.04)	(0.04)
Age	0.00	0.00	0.00
	(0.00)	(0.00)	(0.00)
Rural site	0.03	0.04	0.04
	(0.05)	(0.05)	(0.05)
Married	−0.02	−0.01	−0.00
	(0.05)	(0.05)	(0.05)
Educational background (0 = primary school and below)			
Junior high school	0.17 *	0.14 *	0.14 *
	(0.07)	(0.07)	(0.07)
Senior high school	0.26 **	0.24 **	0.24 **
	(0.09)	(0.08)	(0.08)
Associate college and above	0.15	0.13	0.13
	(0.10)	(0.10)	(0.10)
Annual household income (0 = less than 40,000 RMB)			
40,000–59,999 RMB	0.05	0.05	0.04
	(0.06)	(0.06)	(0.06)
60,000–89,999 RMB	−0.02	−0.04	−0.04
	(0.07)	(0.07)	(0.07)
90,000 RMB and above	−0.00	−0.02	−0.02
	(0.08)	(0.08)	(0.08)
Earthquake exposure			
Being buried	0.03	0.03	0.03
	(0.14)	(0.13)	(0.13)
Being injured	−0.07	−0.06	−0.07
	(0.06)	(0.06)	(0.06)
Being disabled	−0.03	−0.05	−0.05
	(0.16)	(0.16)	(0.15)
Family died	−0.10	−0.11	−0.12
	(0.09)	(0.09)	(0.09)
Family injured	−0.01	−0.02	−0.02
	(0.06)	(0.06)	(0.06)
Family disabled	−0.03	−0.01	−0.01
	(0.09)	(0.09)	(0.09)
Kinsfolk died	−0.04	−0.04	−0.03
	(0.06)	(0.06)	(0.06)
Kinsfolk injured	−0.05	−0.05	−0.06
	(0.05)	(0.04)	(0.04)
Kinsfolk disabled	−0.02	−0.02	−0.03
	(0.06)	(0.06)	(0.06)
Acquaintance died	−0.06	−0.04	−0.04
	(0.05)	(0.05)	(0.05)
Acquaintance injured	0.01	0.01	0.01
	(0.04)	(0.04)	(0.04)
Acquaintance disabled	0.20 ***	0.19 ***	0.19 ***
	(0.05)	(0.05)	(0.05)
Witness to others’ bury	−0.04	−0.05	−0.05
	(0.06)	(0.06)	(0.06)
Witness to others’ death	−0.01	−0.02	−0.02
	(0.05)	(0.05)	(0.05)
Witness to others’ injury	0.18 ***	0.18 ***	0.17 ***
	(0.05)	(0.05)	(0.05)
Loss of house and property (0 = mildly)			
Moderate	0.12 *	0.12 *	0.12 *
	(0.05)	(0.05)	(0.05)
Serious	0.25 ***	0.25 ***	0.25 ***
	(0.06)	(0.06)	(0.06)
Constant	0.47 +	−1.78 **	−1.76 **
	(0.26)	(0.56)	(0.56)
Observations	1000	1000	1000
DF	30	32	33
chi2	496.7	522.4	526.5
Log likelihood	−961	−948.1	−946
PR2	0.205	0.216	0.218

*** *p* < 0.001, ** *p* < 0.01, * *p* < 0.05, + *p* < 0.1.
